# The effect of carbohydrases or prebiotic oligosaccharides on growth performance, nutrient utilisation and development of small intestine and immune organs in broilers fed nutrient‐adequate diets based on either wheat or barley

**DOI:** 10.1002/jsfa.9537

**Published:** 2019-02-05

**Authors:** Allison D Craig, Mike R Bedford, Peter Hastie, Farina Khattak, Oluyinka A Olukosi

**Affiliations:** ^1^ Monogastric Science Research Centre, Scotland's Rural College Edinburgh EH9 3JG UK; ^2^ McCall Building, School of Veterinary Medicine University of Glasgow Glasgow UK; ^3^ A B Vista, Woodstock Centre Marlborough UK; ^4^ Department of Poultry Science University of Georgia Athens GA USA

**Keywords:** carbohydrases, broilers, prebiotic oligosaccharides, nutrient digestibility

## Abstract

**BACKGROUND:**

Non‐starch polysaccharides are large complex molecules and are found in cereal grains. This study was conducted to investigate the effect of carbohydrase enzymes or prebiotic oligosaccharides on growth performance, nutrient utilisation and weight of organs associated with the immune system in broilers fed wheat‐ or barley‐based diets.

**RESULTS:**

In wheat‐based diets, feed intake was lower (*P* < 0.05) following xylo‐oligosaccharide supplementation, whereas in barley‐based diets feed intake was greater (*P* < 0.05) following β‐glucanase supplementation. Gross energy digestibility was improved (*P* < 0.01) when either level of xylanase was added to wheat diets. Ileal digestible energy was greater (*P* < 0.01) in wheat diets including an additive compared with the control diet. In wheat diets, bursa weight was lower (*P* < 0.05) following xylo‐oligosaccharide supplementation compared with the control treatment.

**CONCLUSIONS:**

The current study showed that supplemented carbohydrases or prebiotic oligosaccharides could alter the development of immune organs or small intestine without any significant effect on growth performance in broilers receiving nutrient‐adequate diets. © 2018 The Authors. *Journal of the Science of Food and Agriculture* published by John Wiley & Sons Ltd on behalf of Society of Chemical Industry.

## INTRODUCTION

Non‐starch polysaccharides (NSPs) are large complex molecules found in cereal grains commonly used in poultry diets. NSPs are not digested by endogenous enzymes produced by broilers resulting in several anti‐nutritive effects. The main effect of NSPs is an increase in digesta viscosity^1^ which has a negative impact on bird performance and nutrient utilisation. The second effect of NSPs is referred to as the cage effect, whereby nutrients are trapped within the large structure of NSPs meaning that they are inaccessible to enzymes for digestion.^2^ The amount and type of NSPs can differ between cereals meaning that different cereal grains can have differing effects on growth and nutrient utilisation. NSPs also affect the way in which the gastrointestinal tract develops. In diets high in NSPs, the small intestine increases in size in order to accommodate the increase in viscosity and produce the volume of secretions required to digest it.^3^

To combat these effects, carbohydrase enzymes are added to poultry diets. Carbohydrases are able to hydrolyse NSPs, decreasing digesta viscosity and releasing entrapped nutrients, improving growth performance and nutrient utilisation.^4^ Carbohydrases also affect the way in which the small intestine develops; for example, xylanase has been shown to decrease the length of the small intestine.^3^ In addition to this, there is evidence that carbohydrases produce small oligosaccharides during hydrolysis of NSPs that may have prebiotic properties.^5^

A prebiotic is defined as ‘a non‐digestible food ingredient that beneficially affects the host by selectively stimulating the growth and/or activity of one or a limited number of bacteria in the colon and thus improves host health’.^6^ There are many oligosaccharides that can be considered as having prebiotic properties. Two of these are xylo‐oligosaccharide (XOS) and galacto‐oligosaccharide (GOS) which have both been shown to increase the population of *Bifidobacterium* in the gut of broiler chickens.

The current study was designed to investigate the effect of carbohydrases and prebiotic oligosaccharides on growth, nutrient utilisation and immune responses in broilers fed diets based on different cereal grains.

## MATERIALS AND METHODS

### Animals, diets and housing

All the procedures in the experiment were approved by the SRUC Animal Experiment Committee.

In total, 384 Ross 308 broilers were used for this experiment. On arrival (day 0), the birds were weighed and allocated to one of eight dietary treatments with eight birds per pen and six replicates per treatment. The study followed a randomised complete block design. The treatments were arranged into a 2 × 4 factorial arrangement with two diet types (wheat‐ or barley‐based) and four additive types (no additive, carbohydrases at 16 000 or 32 000 XU kg^−1^ or prebiotic). Wheat‐based diets were supplemented with xylanase (Econase XT, AB Vista, Marlborough, UK) or XOS (Shandong Lifelong Bio‐technology Co., China). The xylanase used contained 160 000 units of endo‐1,4‐β‐xylanase activity. One unit of xylanase activity is defined as the amount of enzyme required to liberate 1 nmol of reducing sugars from xylan using a standardised test. Barley‐based diets were supplemented with β‐glucanase (Econase GT, AB Vista, Marlborough, UK) or GOS (Vivinal GOS powder, The Netherlands). The β‐glucanase used contained 160 000 units of endo‐1,3,4‐β‐glucanase activity. One unit of β‐glucanase activity is defined as the amount of enzyme required to liberate 1 nmol of reducing sugars from β‐glucan using a standardised test. All of the diets were formulated to meet the nutrient requirements of the birds. The compositions of basal diets are presented in Table 1.

**Table 1:** The ingredients and analysed nutrient composition (g kg^−1^) of wheat‐ and barley‐based control diets fed to broilers from day 0 to day 22 post hatch

Items (g kg^−1^)	Diet 1 (wheat‐based control)	Diet 2 (barley‐based control)
Wheat	461.3	100.0
Wheat bran	80.0	52.0
Corn	29.5	20.0
Wheat germ	86.5	100.0
Barley	0.00	344.5
Soybean meal	230.0	261.0
Soya oil	40.0	50.0
Salt	1.0	2.0
Limestone	13.3	14.0
Dicalcium phosphate (18% P)	13.0	11.1
Sodium bicarbonate	5.0	3.5
Lysine HCl	5.0	2.0
Methionine	3.0	2.5
Threonine	2.0	2.0
Vitamin and trace mineral premix	5.0	5.0
Titanium dioxide premix	27.0	27.0
Analysed nutrient (g kg^−1^)		
Dry matter	905.0	899.0
Gross energy (MJ kg^−1^)	18.77	20.03
Cl	1.1	1.8
Na	1.5	1.4
Ca	9.4	8.9
P	6.0	5.7
N	34.5	34.4

The vitamin and trace minerals premix provided (units kg^−1^ diets): retinol 16 000 iu; cholecalciferol 33 000 iu; tocolpherol 75 iu; thiamine 3 mg; riboflavin 10 mg; pyridoxine 3 mg; cyanocobalamin 15 μg; phylloquinone 5 mg; nicotinic acid 60 mg; pantothenic acid 14.5 mg; folic acid 1.5 mg; biotin 275 μg; choline chloride 250 mg; iron 20 mg; copper 10 mg; m100 mg; cobalt 1 mg; zinc 82 mg; iodine 1 mg; selenium 0.2 mg; molybdenum 0.5 mg.

The birds were kept in raised floor pens with wire floors which were covered with cardboard and shavings for the first 19 days. On day 20 the cardboard and shavings were removed to enable excreta collection. Feed and water were provided on an *ab libitum* basis for the duration of the trial (day 0 until day 22).

### Sample collection

On day 21 the feed and birds were weighed. Two birds from each pen were euthanised by overdose of barbiturate and weighed individually. The length and weight of duodenum, jejunum and ileum sections and weight of empty gizzard, spleen and bursa were recorded. The remaining birds were euthanised on day 22 and ileal digesta was collected.

### Weight of empty gizzard, spleen and bursa

The gizzard, spleen and bursa were removed from two birds per pen. The gizzard was emptied leaving the yellow lining intact and all three organs were weighed to give an indication of the development of the gut and organs associated with the immune system.

### Gastrointestinal tract length and weight

The duodenum, jejunum and ileum of two birds per pen were cut into sections following the method set out by Olukosi *et al*.^7^ The duodenum is defined as the small intestine section spanning the pancreatic loop. The jejunum is defined as the small intestine section from the pancreatic loop to Meckel's diverticulum, whereas the ileum is defined as the small intestine section from Meckel's diverticulum to the ileocecal junction. The small intestine sections from both birds were flushed with saline solution and the length and weight of each small intestine section were recorded.

### Growth performance

Weights of birds and feed were recorded on day 0 and day 21 and used to calculate feed intake and feed conversion ratio per pen. Excreta were collected on days 20 and 21 while ileal digesta was collected on day 22.

### Chemical analysis

The determination of titanium in diet, digesta and excreta samples was carried out according to the method of Short *et al*.^8^ Dry matter (DM) and nitrogen determinations were done using standard methods from the AOAC.^9^ DM was determined by drying 1 g of a sample in a unitherm forced draft drying oven (Unitherm, Russel‐Lindsay Engineering Ltd, Birmingham, UK) at 95 °C for 24 h (Method 934.01; AOAC^9^). Nitrogen determination was carried out using the combustion method (Method 968.06, AOAC^9^). Gross energy was determined using an isoperibol bomb calorimeter system using benzoic acid as an internal standard (model 6200, Parr Instruments, Moline, Illinois, USA). Total tract retention and ileal digestibility were determined using the index method as described by Olukosi *et al*.^10^

### Statistics

Statistical analysis was done using the ANOVA function of Genstat (14th Edition). Data were analysed following a 2 × 4 factorial arrangement with body weight as the blocking factor. When an interaction between diet type and additive type was identified the means were separated using specific contrasts. Main effect means were separated using a Fisher multiple comparisons test. Significance was set at *P* ≤ 0.05 and tendencies were declared at 0.05 < *P* < 0.1.

## RESULTS

### Growth performance

The growth performance data are presented in Table 2. The feed intake was significantly affected by a diet type × additive type interaction. The feed intake of birds receiving the wheat diet supplemented with XOS was lower (*P* < 0.05) than that of the birds receiving the wheat control diet but the addition of xylanase had no effect. The feed intake of birds receiving the barley‐based diet supplemented with β‐glucanase at 16 000 U (*P* < 0.05) and 32 000 U was greater (*P* < 0.05) than that of the barley control; however, the addition of GOS had no significant effect. The body weight gain of broilers was greater (*P* < 0.05) in diets containing 16 000 or 32 000 U kg^−1^ of enzyme compared to the control or oligosaccharide treatments. There were no effects on feed conversion ratio.

**Table 2:** The growth performance response of broilers fed diets supplemented with carbohydrases or prebiotic oligosaccharides

Diet type	Additive	BWG (g)	FI (g)	FCR
Simple effect means				
Wheat	Control (1)	771.2	1174	1.53
	Xylanase 16 000 U kg^−1^ (2)	805.6	1137	1.41
	Xylanase 32 000 U kg^−1^ (3)	832.5	1130	1.39
	XOS (4)	801.4	1090	1.38
Barley	Control (5)	755.2	1084	1.45
	β‐Glucanase16000 U kg^−1^ (6)	835.9	1155	1.38
	β‐Glucanase 32 000 U kg^−1^ (7)	815.85	1146	1.45
	GOS (8)	745.72	1117	1.51
Pooled SEM		23.4	20.1	0.044
Means for main effect of diet type				
Wheat		802.7	1133	1.43
Barley		788.2	1126	1.45
Pooled SEM		11.7	14.2	0.022
Means for main effect of additive type				
	None	763.2^a^	1129	1.49
	16 000 U kg^−1^	820.7^bc^	1146	1.40
	32 000 U kg^−1^	824.2^c^	1138	1.42
	Oligosaccharide	773.6^ab^	1104	1.45
Pooled SEM		16.5	10.1	0.031
*P* values for main effects and interaction				
Diet type		0.387	0.623	0.489
Additive		0.023	0.192	0.235
Diet type × additive		0.351	0.017	0.091
*P*‐values for contrasts				
1 *vs* 2			0.192	
1 *vs* 3			0.128	
1 *vs* 4			0.005	
5 *vs* 6			0.017	
5 *vs* 7			0.036	
5 *vs* 8			0.246	

BWG, body weight gain; FI, feed intake; FCR, feed conversion ratio; XOS, xylo‐oligosaccharide; GOS, galacto‐oligosaccharide; means within the same column and diet type with different superscripts are significantly different (*P* < 0.05).

### Nutrient digestibility and total tract retention

The ileal digestibility data are summarized in Table 3. There was a diet type × additive type interaction for DM. DM was greater (*P* < 0.01) when 32 000 U kg^−1^ of xylanase was added to wheat‐based diets compared with wheat control. DM was lower (*P* < 0.01) when XOS was added to wheat‐based diets compared with the wheat control. In barley diets, DM was lower (*P* < 0.01) when 16 000 U kg^−1^ of β‐glucanase was added and greater (*P* < 0.01) when GOS was added compared with barley control. There was a significant diet type × additive type interaction for gross energy digestibility. Gross energy digestibility was greater (*P* < 0.05) in wheat diets when either level of xylanase was added; however, no effect was seen in barley diets. There was a significant diet type × additive type interaction for N. In barley diets, N was lower (*P* < 0.05) when 16 000 U kg^−1^ of β‐glucanase was added compared with barley control. There was a significant diet type × additive type interaction for ileal digestible energy (IDE). In wheat diets, IDE was greater (*P* < 0.01) when any of the additives were used compared with wheat control. In barley diets, IDE was lower (*P* < 0.01) when GOS was added compared with barley control.

**Table 3:** Coefficients of ileal digestibility of nutrients in broilers fed diets supplemented with carbohydrases or prebiotic oligosaccharides

Diet type	Additive	DM	Gross energy	N	IDE (MJ kg^−1^)
Simple effect means					
Wheat	Control (1)	0.70	0.73	0.73	13.63
	Xylanase16000 U kg^−1^ (2)	0.71	0.76	0.74	15.92
	Xylanase 32 000 U kg^−1^ (3)	0.73	0.76	0.76	14.28
	XOS (4)	0.69	0.73	0.72	14.39
Barley	Control (5)	0.67	0.72	0.71	14.38
	β‐Glucanase 16 000 U kg^−1^ (6)	0.63	0.70	0.67	14.63
	β‐Glucanase 32 000 U kg^−1^ (7)	0.66	0.72	0.70	14.71
	GOS (8)	0.72	0.72	0.71	12.70
Pooled SEM		0.009	0.008	0.011	0.148
Means for main effect of diet type					
Wheat		0.71	0.74	0.74	14.56
Barley		0.67	0.71	0.70	14.10
Pooled SEM		0.004	0.004	0.006	0.074
Means for main effect of additive type					
	None	0.69	0.72	0.72	14.00
	16 000 U kg^−1^	0.67	0.73	0.70	15.27
	32 000 U kg^−1^	0.70	0.74	0.73	14.50
	Oligosaccharide	0.71	0.73	0.71	13.55
Pooled SEM		0.006	0.005	0.008	0.105
*P* values for main effects and interaction					
Diet type		<0.001	<0.001	<0.001	<0.001
Additive		<0.001	0.354	0.095	<0.001
Diet Type × additive		<0.001	0.010	0.021	<0.001
*P* values for contrasts					
1 *vs* 2		0.594	0.012	0.823	<0.001
1 *vs* 3		0.013	0.012	0.053	0.004
1 *vs* 4		0.568	0.645	0.448	0.001
5 *vs* 6		0.001	0.105	0.008	0.239
5 *vs* 7		0.457	0.900	0.315	0.123
5 *vs* 8		<0.001	0.733	0.877	<0.001

DM, dry matter; IDE, ileal digestible energy; XOS, xylo‐oligosaccharide; GOS, galacto‐oligosaccharide; means with different subscripts within the same column and diet type are significantly different (*P* < 0.05).

Total tract retention data is presented in Table 4. DM was significantly affected by a diet type × additive type interaction. DM was greater (*P* < 0.01) when 32 000 U kg^−1^ of xylanase was added to wheat diets compared with the wheat control. DM was lower (*P* < 0.01) when 16 000 U kg^−1^ of B‐glucanase was added to barley diets and greater (*P* < 0.01) when GOS was added to barley diets compared with the barley control. Gross energy retention was greater (*P* < 0.05) when either 16 000 or 32 000 U kg^−1^ of xylanase was added to wheat diets compared with the wheat control diet. Gross energy retention was greater (*P* < 0.05) in wheat‐based diets compared to barley‐based diets. Nitrogen digestibility was greater (*P* < 0.05) when 32 000 U kg^−1^ of carbohydrase or prebiotic oligosaccharide was added to the diet compared with supplementing diets with 16 000 U kg^−1^ of carbohydrase.

**Table 4:** Coefficients of total tract retention of nutrients from broilers fed diets supplemented with carbohydrases or prebiotic oligosaccharides

Diet type	Additives	DM	Gross energy	N
Simple effect means				
Wheat	Control (1)	0.70	0.72	0.68
	Xylanase 16 000 U kg^−1^ (2)	0.71	0.76	0.66
	Xylanase 32 000 U kg^−1^ (3)	0.73	0.76	0.71
	XOS (4)	0.69	0.73	0.66
Barley	Control (5)	0.67	0.72	0.69
	β‐Glucanase 16 000 U kg^−1^ (6)	0.63	0.70	0.62
	β‐Glucanase 32 000 U kg^−1^ (7)	0.66	0.72	0.69
	GOS (8)	0.72	0.72	0.67
Pooled SEM		0.008	0.007	0.012
Means for main effect of diet type				
Wheat		0.71	0.73	0.68
Barley		0.67	0.71	0.67
Pooled SEM		0.004	0.004	0.006
Means for main effect of additive type				
	None	0.69	0.70	0.68^bc^
	16 000 U kg^−1^	0.67	0.72	0.64^a^
	32 000 U kg^−1^	0.70	0.74	0.70^c^
	Oligosaccharide	0.71	0.73	0.67^b^
Pooled SEM		0.006	0.005	0.008
*P*‐values for main effects and interaction				
Diet type		<0.001	<0.001	0.162
Additive		<0.001	0.179	<0.001
Diet type × additive		<0.001	0.004	0.116
*P*‐vales for contrasts				
1 *vs* 2		0.430	0.004	
1 *vs* 3		0.005	0.004	
1 *vs* 4		0.710	0.438	
5 *vs* 6		<0.001	0.102	
5 *vs* 7		0.382	0.894	
5 *vs* 8		<0.001	0.537	

DM, dry matter; N, nitrogen content; XOS, xylo‐oligosaccharide; GOS, galacto‐oligosaccharide; means with different subscripts within the same diet type and column are significantly different (*P* < 0.05).

### Organ weight

The organ weight data were analysed relative to individual body weight and are summarized in Table 5.The weight of the bursa relative to body weight shows a diet type × additive type interaction (*P* < 0.05) whereby birds receiving the wheat control diet had greater relative bursa weight than those receiving the wheat plus XOS diet. There was a significant effect of additive type (*P* < 0.05) observed for relative gizzard weight: the addition of oligosaccharides to the diet reduced relative gizzard weight compared with control. There was also a main effect of diet type on relative gizzard weight. In wheat‐based diets, relative gizzard weight was lower (*P* < 0.05) than that in barley‐based diets. There was a tendency for a diet type effect (*P* = 0.076) on relative spleen weight as all treatments increased relative spleen weight compared with control.

**Table 5:** Empty gizzard, spleen and bursa of fabricius weight relative to individual body weight (g kg^−1^) of broilers fed diets supplemented with carbohydrases or prebiotic oligosaccharides

Diet type	Additive	Relative gizzard weight	Relative spleen weight	Relative bursa weight
Simple effect means				
Wheat	Control (1)	23.3	0.8	2.2
	Xylanase 16 000 U kg^−1^ (2)	22.9	0.8	2.5
	Xylanase 32 000 U kg^−1^ (3)	21.8	0.9	2.4
	XOS (4)	20.2	0.9	1.8
Barley	Control (5)	25.5	0.9	2.6
	β‐Glucanase 16 000 U kg^−1^ (6)	24.1	1.1	2.0
	β‐Glucanase 32 000 U kg^−1^ (7)	23.7	0.9	2.2
	GOS (8)	21.8	1.0	2.1
Pooled SEM		1.09	0.10	0.15
Means for main effect of diet type				
Wheat		22.0	0.8	2.2
Barley		23.8	1.0	2.2
Pooled SEM		0.54	0.05	0.07
Means for main effect of additive type				
	None	24.4^b^	0.8	2.4
	16 000 U kg^−1^	23.5^ab^	0.9	2.2
	32 000 U kg^−1^	22.7^ab^	0.9	2.3
	Oligosaccharide	21.0^a^	1.0	2.0
Pooled SEM		0.77	0.07	0.10
*P*‐values for main effects and interaction				
Diet type		0.031	0.076	0.744
Additive		0.027	0.717	0.028
Diet type × additive		0.976	0.360	0.009
*P*‐values for contrasts				
1 *vs* 2				0.206
1 *vs* 3				0.456
1 *vs* 4				0.041
5 *vs* 6				0.178
5 *vs* 7				0.672
5 *vs* 8				0.547

Data were analysed relative to individual body weight. XOS, xylo‐oligosaccharide; GOS, galacto‐oligosaccharide; means with different subscripts within the same diet type and column are significantly different (*P* < 0.05).

### Duodenum, jejunum and ileum length and weight

The length and weight of small intestine sections are presented in Table 6. There was a significant diet type × additive type interaction (*P* < 0.01) for ileum length. Ileum length was lower (*P* < 0.01) for wheat diets when supplemented with 16 000 U kg^−1^ of xylanase. However, ileum length was greater (*P* < 0.01) when barley diets were supplemented with either 16 000 or 32 000 U kg^−1^ of β‐glucanase.

**Table 6:** The length and weight of small intestine sections relative to total small intestine length or weight of broilers fed diets supplemented with carbohydrases or prebiotic oligosaccharides

Diet type	Additive	DW/TSIW	DL/TSIL	JW/TSIW	JL/TSIL	IW/TSIW	IL/TSIL
Simple effect means							
Wheat	Control (1)	166.9	16.58	421.6	40.81	411.5	42.61
	Xylanase 16 000 U kg^−1^ (2)	178.4	17.31	450.7	42.75	371.0	40.58
	Xylanase 32 000 U kg^−1^ (3)	159.5	16.39	446.0	41.99	394.5	41.62
	XOS (4)	164.4	16.45	454.8	41.02	380.8	42.57
Barley	Control (5)	165.9	18.03	445.3	41.22	388.8	40.75
	β‐Glucanase 16 000 U kg^−1^ (6)	170.0	16.61	439.6	41.20	390.4	42.20
	β‐Glucanase 32 000 U kg^−1^ (7)	170.0	16.02	448.5	41.52	381.4	42.46
	GOS (8)	167.8	17.00	440.5	41.57	391.8	41.43
Pooled SEM		6.53	0.611	12.07	0.514	13.56	0.433
Means for main effect of diet type							
Wheat		167.3	16.69	443.3	41.64	389.4	41.85
Barley		168.4	16.91	443.5	41.38	388.1	41.71
Pooled SEM		3.26	0.306	6.04	0.257	6.78	0.261
Means for main effect of additive type							
	Control	166.4	17.31	433.5	41.01	400.1	41.68
	16 000 U kg^−1^	174.2	16.96	445.1	41.97	380.7	41.39
	32 000 U kg^−1^	164.7	16.21	447.3	41.75	338.0	42.04
	Oligosaccharide	166.1	16.73	447.7	41.30	386.3	42.00
Pooled SEM		4.62	0.432	8.54	0.364	9.59	0.306
*P*‐values for main effects and interaction							
Diet type		0.807	0.602	0.981	0.474	0.889	0.655
Additive		0.469	0.348	0.609	0.253	0.541	0.411
Diet type × additive		0.536	0.304	0.393	0.174	0.376	<0.001
*P*‐values for contrasts							
1 *vs* 2							0.002
1 *vs* 3							0.115
1 *vs* 4							0.952
5 *vs* 6							0.025
5 *vs* 7							0.009
5 *vs* 8							0.278

DW, JW and IW were analysed relative to total small intestine weight; DL, JL and IL were analysed relative to total small intestine length. DW, duodenum weight; DL, duodenum length; JW, jejunum weight; JL, jejunum length; IW, ileum weight; IL, ileum length; TSIL, total small intestine length; TSIW, total small intestine weight; XOS, xylo‐oligosaccharide; GOS, galacto‐oligosaccharide; means with different subscripts within the same diet type and column are significantly different (*P* < 0.05).

## DISCUSSION

The aim of the study was to investigate the effect of carbohydrase enzymes or prebiotic oligosaccharides on growth performance and the development of the small intestine and organs associated with the immune system in broilers receiving wheat‐ and barley‐based diets. Wheat diets were supplemented with either xylanase or XOS and barley‐based diets were supplemented with β‐glucanase or GOS.

### Growth performance

The mechanism by which enzymes improve growth performance is through the hydrolysis of NSPs. NSP hydrolysis reduces digesta viscosity, releases encapsulated nutrients and creates potentially prebiotic oligosaccharides which could lead to an improvement in growth performance.^11^ The addition of carbohydrase enzymes or prebiotic oligosaccharides to wheat‐ or barley‐based diets did not significantly affect growth performance in the current study. Although this was not expected, it is not uncommon. The evidence for the use of additives like enzymes or prebiotics in broilers is highly variable.^12^, ^13^, ^14^, ^15^

One of the possible explanations for variation in the effect of carbohydrase enzymes on the growth performance is the composition of the cereals used within broiler diets. The composition of cereal crops varies for many reasons including variety, growing conditions and storage after harvest.^15^, ^16^ Arabinoxylan is the main component of NSPs found in wheat. The degree of branching within its molecular structure could be affected by the composition of cereal crops. Smeets *et al*.^16^ found that some xylanases prefer to cleave highly branched sections of arabinoxylan and speculated that this could be a factor affecting xylanase action in animal trials. The same can be said for barley. The main type of NSP found in barley grains is β‐glucan which can vary in different plants depending on the presence or absence of side chains.^17^ It has been demonstrated that supplementing barley‐based diets with different β‐glucanases can have varying effects on growth performance.^18^ This suggests that the structure of cereal grains used within broiler diets could be a contributing factor to the variation in improvements in growth performance reported in animal trials.

### Nutrient utilisation

Although there were no significant effects on growth performance in this study, nutrient utilisation was significantly affected by diet type. This could be attributed to the different types of NSP found in wheat and barley grains. Although wheat and barley contain different types of NSP, neither type can be hydrolysed by endogenous enzymes in poultry. Arabinoxylan is the most common type of NSP found in wheat,^2^ whereas barley contains a high percentage of β‐glucan.^17^ Arabinoxylan and β‐glucan have both been associated with an increase in digesta viscosity which leads to a decrease in nutrient utilisation. Viscosity is dependent on many factors including the physical properties of cereal grains. One of these factors is molecular weight. β‐Glucan has a greater molecular weight than arabinoxylan which suggests that barley diets will have a greater viscosity than wheat diets.^2^

To reduce digesta viscosity, carbohydrase enzymes are included in broiler diets. The current study agrees with previous work^2^, ^19^ by demonstrating an improvement in IDE and DM utilisation when broiler diets were supplemented with a carbohydrase enzyme or a prebiotic. This was dependent on the type of cereal included in the diet. The addition of carbohydrase enzymes to cereal‐based diets improves nutrient utilisation which is explained through three different mechanisms in the literature. The first describes how carbohydrase enzymes reduce digesta viscosity by hydrolysing NSPs. This allows sufficient mixing of the digesta to increase nutrient absorption and maintain a steady supply of nutrients to the commensal microflora that line the surface of the small intestine.^15^ The second describes the effect of enzymes on the structure of cereal grains which often trap nutrients such as protein and energy. Carbohydrase enzymes hydrolyse the NSPs releasing the trapped nutrients which can then be absorbed in the small intestine.^20^ These released nutrients contribute to the increase in nutrient utilisation often reported following carbohydrase supplementation. The third explains how carbohydrases could increase the solubility of NSPs. The theory explained by Amerah *et al*.^21^ suggests that during NSP hydrolysis carbohydrase enzymes disrupt the packaging of the NSP molecules allowing nutrients that may be trapped to move around more easily, making the molecules more soluble and available for nutrient absorption in the small intestine. It is likely that carbohydrases improve nutrient utilisation through a combination of all three of these mechanisms which may help to explain why nutrient utilisation differs between wheat and barley diets in the current study.

Prebiotic oligosaccharides improve nutrient utilisation in a different way. In order to be classed as a prebiotic, the molecule in question must be undigested when it reaches the intestine and beneficially stimulate the host microflora.^6^ Prebiotics selectively stimulate the growth of beneficial microflora such as Bifido‐bacter and lacto‐bacilli^22^ and discourage the growth of potential pathogens such as *Escherichia coli*. This, in turn, increases the production of short‐chain fatty acids (SCFAs) which can be used by cells of the colon for energy.^22^ The production of SCFAs has an impact on nutrient utilisation in two ways. Firstly, nutrients that would have been used to maintain the immune status of the gut may not be needed anymore following a reduction in potential pathogens and can be directed elsewhere.^22^ Secondly, the cells of the colon may not require as much energy for maintenance following the increased production of SCFAs. However, the evidence for prebiotic oligosaccharides improving nutrient utilisation is inconsistent with some studies disagreeing proposing that prebiotics have any effect on nutrient utilisation.^23^, ^24^

### Organ development

The bursa of fabricius is small immune organ situated above the bird cloaca and is responsible for B cell maturation. The bursal lumen is connected to the gut lumen via the bursal duct which allows fluid containing gut contents and other molecules such as antigens to flow into the bursa of fabricius.^25^ This continuous flow of gut antigens enables the bursal B cells to mature and migrate to the gut lumen.^25^ An increase in antigens from the gut may lead to an increase in bursa of fabricius weight meanwhile a decrease in antigens from the gut may lead to a decrease in bursa of fabricius weight. The current study would agree with this suggestion; however, the effect of prebiotic oligosaccharide differed depending on the type of cereal grain used in the diet. In wheat diets, XOS reduced bursa of fabricius weight compared to the control; however, GOS had no effect on bursa of fabricius weight in barley diets. This was unexpected as both XOS and GOS have been linked to an increase in the same groups of beneficial microbes such as bifido‐ and lacto‐bacilli.^21^, ^22^, ^26^ It has been suggested that the effect of prebiotic oligosaccharides on the microbial population is linked to their molecular structure. The prebiotic oligosaccharides used in the current study contained different linkages: XOS contains β(1‐4) linkages and GOS contains α(1‐4) and β(1‐6) linkages.^27^ The functional properties of these prebiotic oligosaccharides are known to vary depending on the combination of monomers, polymerisation and osidic bonds within their structure.^27^ Perhaps this is why XOS appeared to have a greater effect than GOS on bursa of fabricius weight, which could be an indicator of immune function.

Whilst bursa of fabricius weight is affected by the chemical properties of the diet, gizzard weight is often affected by the physical properties of the diet. The gizzard is a muscular organ used to grind feed to an appropriate particle size before it progresses to be digested and nutrients absorbed in the small intestine.^28^ Gizzard weight was significantly lower in wheat‐based diets compared to barley‐based diets in the current study. In previous studies increases in gizzard weight have been associated with the use of wholegrains in broiler diets.^28^ The reason for the increase in gizzard weight is that a larger muscle mass is required, as well as longer retention time, to grind down larger particles before they can leave the gizzard, increasing gizzard weight.^29^, ^30^ It is likely that the increase in gizzard weight in barley‐based diets compared to wheat‐based diets is due to an increase in fibre in barley grain compared with wheat grain.^2^

In the current study, a decrease in gizzard weight was also observed when prebiotic oligosaccharide was added to the diet compared with the control. Once digesta leaves the gizzard and enters the proventriculus, digestive enzymes and acid are released and mixed with the digesta to begin the process of chemical digestion. Some of the digesta mixed with acid and enzymes can reflux back into the gizzard.^28^ Morgan *et al*.^31^ showed that the highest conversion of arabinoxylan to XOS was at pH 2.5 which is more likely to be found in the gizzard or crop. The mixing of gizzard content with the acidic solution found in the proventriculus could activate the carbohydrase enzymes and kick‐start NSP hydrolysis, resulting in a reduction in digesta viscosity and a decrease in gizzard weight. Prebiotic oligosaccharides, however, are not known to alter the physical properties of the diet and have been shown to have no effect on gizzard weight in other studies.^32^ However, it has been suggested that changes in villi height and crypt depth in the small intestine can influence increases in gizzard weight^33^ which is also related to retention time. Prebiotic oligosaccharides, such as XOS and GOS, are fermented by bacteria in the small intestine producing SCFAs. The SCFAs stimulate a hormone response, prolonging retention time in the gizzard and causing the gizzard to become larger.^34^

### Duodenum, jejunum and ileum length and weight

The current study did not investigate villi height or crypt depth in the small intestine but did investigate the effect of carbohydrase enzymes and prebiotic oligosaccharides on gross morphology such as intestine length and weight. In the current study, enzyme supplementation decreased the length of the ileum in wheat‐based diets, but in the barley‐based diet ileum length increased following enzyme addition. Broiler diets containing viscous cereals, for example wheat and barley, have been associated with increased cell proliferation and decreased nutrient utilisation in the small intestine.^35^ The inclusion of carbohydrase enzymes reduces digesta viscosity which reduces the abrasive effect of viscous diets resulting in decreased gut length.^36^ The current study would agree with this; however, in barley diets gut length increased in response to carbohydrase enzyme supplementation. One reason for this could be an increase in gut fill as reported by Yaser and Forbes.^37^ If it is assumed that activation of enzyme activity occurs in the gizzard and not the small intestine, the smaller particle size could increase passage rate causing the small intestine to expand to accommodate the extra volume. However, the optimal pH for enzyme action differs from enzyme to enzyme. In this case, xylanase would function at the lower pH found in the gizzard; however, β‐glucanase functions best at a pH of around 4.5–6.5 which is found in the small intestine thus making increased gut fill an unlikely explanation for increased small intestine length in the case of barley‐based diets. Reports on the effect of enzyme supplementation on length and weight of digestive tract are equivocal. Some authors have reported no change in gut length or weight.^38^, ^39^ The reason for this could be linked to the development of the gizzard. Banfield *et al*.^40^ and Wu *et al*.^39^ indicated that birds with well‐developed gizzards may not need to modify the length or weight of the small intestine assuming that the gizzard grinds the feed particles small enough for effective digestion and absorption of nutrients.

## CONCLUSIONS

The supplementation of nutrient‐adequate wheat or barley diet with appropriate carbohydrase enzymes or prebiotic oligosaccharides significantly improved nutrient utilisation at higher supplemented levels but had little effect on growth performance. In addition, the data obtained demonstrate the potential use of carbohydrase enzymes or prebiotic oligosaccharides to influence the development of organs associated with the immune system and the small intestine even with minimal growth performance effects.
